# Bear in mind! Bear presence and individual experience with calf survival shape the selection of calving sites in a long‐lived solitary ungulate

**DOI:** 10.1002/ece3.11177

**Published:** 2024-03-19

**Authors:** Lisa Dijkgraaf, Fredrik Stenbacka, Joris P. G. M. Cromsigt, Göran Ericsson, Wiebke Neumann

**Affiliations:** ^1^ Department of Wildlife Ecology and Conservation Wageningen University (WUR) Wageningen The Netherlands; ^2^ Department of Wildlife, Fish, and Environmental Studies Swedish University of Agricultural Sciences (SLU) Umea Sweden

**Keywords:** anti‐predator behavior, calf survival, glmmTMB, habitat selection, site fidelity, step selection functions

## Abstract

The careful selection of ungulate calving sites to improve offspring survival is vital in the face of predation. In general, there is limited knowledge to which degree predator presence and prey's individual experience shape the selection of calving sites. Predator presence influences the spatiotemporal risk of encountering a predator, while individual experiences with previous predation events shape perceived mortality risks. We used a multi‐year movement dataset of a long‐lived female ungulate (moose, *Alces alces*, *n* = 79) and associated calf survival to test how predator presence (i.e., encounter risk) and females' individual experiences with previous calf mortality events affected their calving site selection and site fidelity. Using data from areas with and without Scandinavian brown bear (*Ursus arctos*) predation, we compared females' calving site selection using individual‐based analyses. Our findings suggest two things. First, bear presence influences calving site selection in this solitary living ungulate. Females in areas with bears were selected for higher shrub and tree cover and showed lower site fidelity than in the bear‐free area. Second, the individual experience of calf loss changes females' selection the following year. Females with lost calves had a lower site fidelity compared to females with surviving calves. Our findings suggest that increased vegetation cover may be important for reducing encounter risk in bear areas, possibly by improving calf concealment. Lower site fidelity might represent a strategy to make the placement of calving sites less predictable for predators. We suggest that bear presence shapes both habitat selection and calving site fidelity in a long‐lived animal, whereas the effect of individual experience with previous calf loss varies. We encourage further research on the relevance of female experience on the success of expressed anti‐predator strategies during calving periods.

## Graphical abstract

Bear presence influences the calving site selection in a solitary living ungulate like moose (*Alces alces*). Females' individual experience of calf loss changes their selection for a calving site the following year. Reduced calving site fidelity in bear areas, relative to the area without bears, might represent a female strategy to make the placement of calving sites less predictable for predators. Increased vegetation cover seems to be an important habitat feature for reducing calf predation risk, possibly by improving calf concealment.

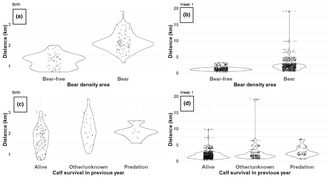

## 1 INTRODUCTION

Predators influence the foraging behavior, habitat choice, survival, and reproductive success of herbivores both directly through eating them and indirectly through predation risk (Gehr et al., 2018; Lima & Dill, 1990; Moll et al., 2017; Preisser & Bolnick, 2008; Say‐Sallaz et al., 2019). Perceived risk of predation may generate considerable costs for prey (Creel & Christianson, 2008) as it, for example, influences prey behavior such as reducing feeding rates and promoting the selection of habitats with reduced predation risk but also reduced food availability (Preisser & Bolnick, 2008). Most mammals and birds possess good spatial memory, which shapes their movement ecology (Kashetsky et al., 2021). Spatial memory can lead to space use patterns such as site fidelity, including the re‐visiting of attractive habitats (Rheault et al., 2021), as well as the avoidance of risky ones (Bracis & Wirsing, 2021). Prey's anti‐predator space use behavior can variously help prey mitigate the spatiotemporal risk of encountering a predator as well as the conditional risk of getting killed when encountering the predator (Lima & Dill, 1990; Moll et al., 2017).

Anti‐predator responses can include both innate and learned behavior (Amo et al., 2011; Berger et al., 2001; Chamaillé‐Jammes et al., 2014; Lewis et al., 2021; Steindler & Letnic, 2021). Innate behavior may reflect responses that have evolved under predator presence for a long time, whereas learned behavior may reflect fine‐tuned responses due to experiences related to individuals surviving potential predation events after encountering a predator. We know that both olfactory and auditory predator cues generate innate anti‐predator responses in mammal and bird species. For example, African herbivores respond to hearing lion (*Panthera leo*) vocalization (Makin et al., 2019), black‐tailed deer respond to wolf odor (Chamaillé‐Jammes et al., 2014), and great tits (*Parus major*) avoid nest boxes with predator odor (Amo et al., 2011). Yet, anti‐predator responses can be context‐specific and be shaped both by species traits and ecosystem features, as well as by changing prevailing environmental conditions (Moll et al., 2017). For example, in areas with expanding or re‐colonizing predator populations, naïve prey species may first need to be exposed to predators (i.e., individual experience) to develop anti‐predator behaviors (Berger et al., 2001; Steindler & Letnic, 2021). Individual experiences can result in learning, as the process of acquiring information through experiences over time can result in changes in neurophysiology and/or behavior (Lewis et al., 2021). Learning and animal movement are closely linked because spatial memory shapes individuals' movement decisions. Movement, as the exploration of space and habitats, offers learning opportunities and, in turn, learning may result in a given set of movement decisions for a given individual (Lewis et al., 2021). Animals can learn by observing others (i.e., social learning) or interactions and experiences with their environment (i.e., individual learning). Individual learning is mostly associative learning in which the animal makes a positive or negative association between a stimulus and the outcome (Lewis et al., 2021). Here, we expect that both predator presence and individual experiences influence anti‐predator behaviors in prey, especially in long‐lived species such as many ungulates.

In ungulates, neonates are especially vulnerable to predation due to their low mobility during their first weeks of life (Swenson et al., 2007). The selection of birth sites, therefore, plays an important role in females' reproductive success. The avoidance of risky places is vital (e.g., the likelihood of predator encounter and the calf being killed), because the reduced mobility of neonates makes the avoidance of risky times more difficult (e.g., the distance to the nearest predator; Moll et al., 2017). To reduce the spatial predation risk for neonates, female ungulates apply different movement strategies, such as erratic movements just before giving birth (Bowyer et al., 1999), selection of specific habitat features of calving sites (Poole et al., 2007) and changes in site fidelity (Testa et al., 2000). In addition to predation risk, however, female ungulates have to consider the availability of sufficient nutrients when selecting calving sites to handle the high‐energy costs of lactation (Bongi et al., 2008; Ciuti et al., 2005; Severud et al., 2019). Thus, the trade‐off between predation risk and food quality promotes the selection of habitats that ensure both low risk and high food quality. For example, ungulate browsers can benefit from high shrub cover as forage but also for concealing their calf from predators (Bongi et al., 2008; Severud et al., 2019; White & Berger, 2001). Fallow deer select areas with greater cover during the calving season (Ciuti et al., 2005). Female ungulates may also select calving sites closer to roads, as has been shown in Yellowstone National Park, which may represent an anthropogenic shield against human‐avoiding bears (Berger, 2007). The success of calf survival during previous years may influence calving site fidelity, where unsuccessful outcomes will result in switching sites due to individual experiences (Morrison et al., 2021; Testa et al., 2000).

Few have empirically studied the influence of both predator presence and individual experiences on ungulate anti‐predator responses in natural settings with different predation risks (but see, Makin et al., 2019; and Thurfjell et al., 2017 for an example of humans as predator). Advancing our knowledge of how observed anti‐predator strategies link to individual experiences in calf survival, and whether such strategies differ between areas with and without predators, will improve our understanding of the plasticity of anti‐predator behavior in a long‐lived species. This becomes especially relevant for prey population dynamics in areas with re‐colonizing or expanding large predator populations, such as brown bears and wolves, in European anthropogenic landscapes (Chapron et al., 2014).

Using multi‐year data on 79 adult female moose (*Alces alces*) from areas with and without brown bears (*Ursus arctos*) across Sweden, we investigated anti‐predator strategies. Specifically, we studied how bear presence and moose female individual experiences (i.e., survival or loss of her calf during the previous year) affected females' choice of habitat features and fidelity to calving sites using high‐resolution individual movement data and calf survival data. We tested the following predictions:

1. Females in bear areas select more strongly for habitat features (shrub and tree cover, terrain ruggedness, and distance to roads) that we expect to reduce perceived bear encounters compared to females in bear‐free areas.
2. Females that experienced calf loss in the previous year select more strongly for these habitat features compared to females with surviving calves to reduce the experienced mortality risk of their neonates.
3. Females that experienced calf predation in the previous year reduce their site fidelity compared to females with surviving calves to reduce the experienced risk of their neonate being killed by a bear.

## 2 METHODS

### 2.1 Study areas

**FIGURE 1:**
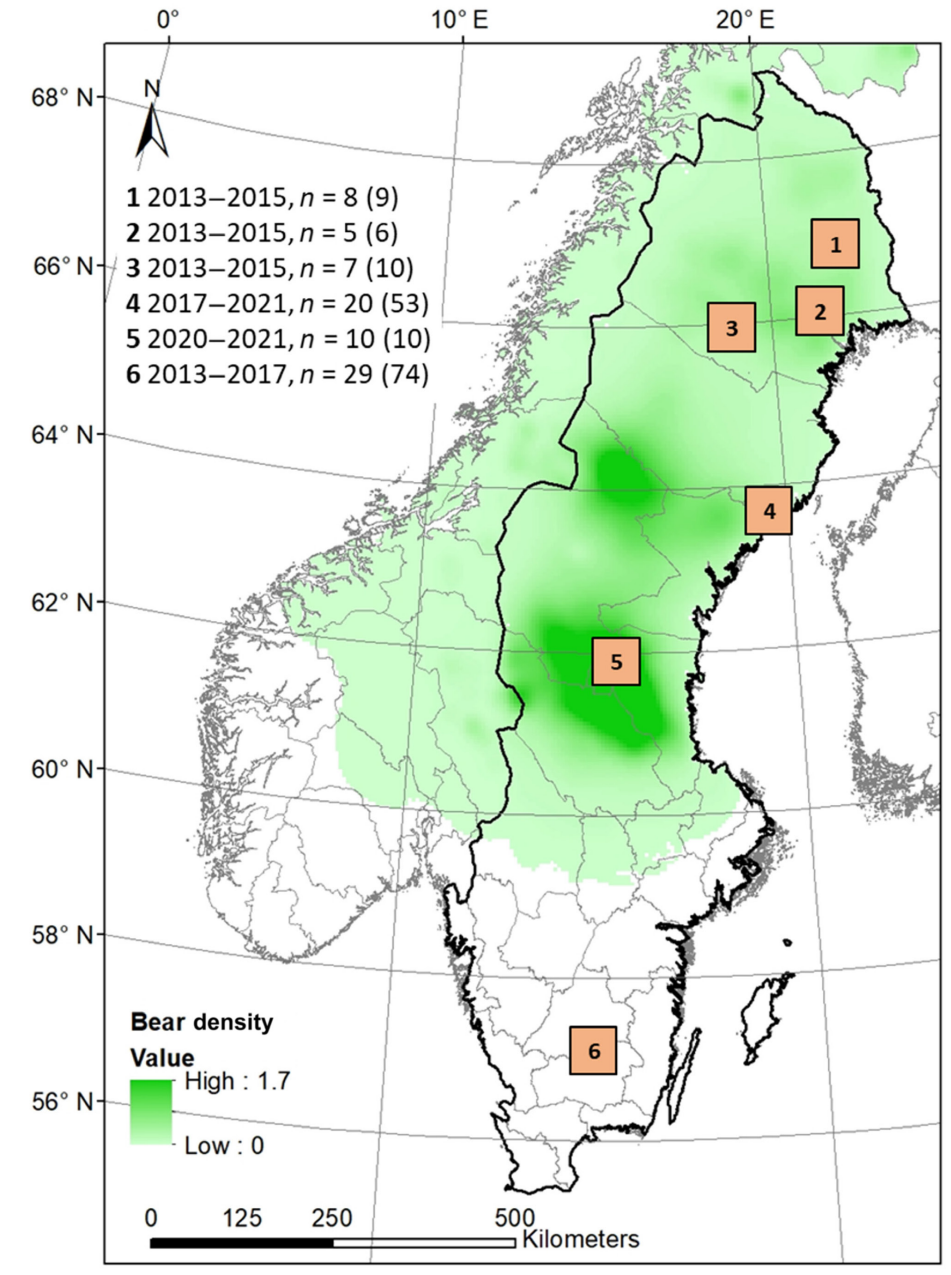
Distribution of the study sites and number of females (year‐moose combinations in parentheses) in relation to estimates on bear density (in 2017) given by Bischof et al. (2020). Sweden in black. Provinces in gray. Study sites in orange. Moose data (*n* = 79 females) were collected between 2013 and 2021. Average bear density: site 1 = 0.12 bears/km^2^ (0.09–0.21), site 2 = 0.19 bears/km^2^ (0.09–0.21), site 3 = 0.17 bears/km^2^ (0.12–0.21), site 4 = 0.04 bears/km^2^ (0–0.10), site 5 = 1.20 bears/km^2^ (0.72–1.60), site 6 = no bears.

We monitored adult free‐ranging female moose and the survival of their calves over multiple years (2013–2021) in six study sites located in the Swedish provinces of Norrbotten, Västerbotten, Gävleborg and Kronoberg (Figure 1). All northern sites (>latitude 61° N) belonged to the northern boreal region, dominated by coniferous forests with Scots pine (*Pinus sylvestris*) and Norway spruce (*Picea abies*), interspersed with mires and patches of deciduous trees (mostly birch (*Betula* spp.) and aspen (*Populus tremula*)). The southern site is placed in the southern boreal region and is dominated by coniferous forests and mixed‐deciduous forests (with deciduous species such as birch, elm (*Ulmus glabra*), oak (*Quercus robur*), maple (*Acer platanoides*) and beech (*Fagus sylvatica*)), interspersed with agricultural fields. All our study sites occurred on flat to gently rolling terrain. In 2005 and 2007, two storms caused major wind throws of coniferous forests in the southern region, generating a large area of regenerating young deciduous and coniferous forests (Valinger et al., 2019). On average, road densities are lower in the northern sites compared to the southern site, whereas bears occur only in the northern part of Sweden. Brown bears have never been extinct in Northern Sweden, but the bear population has changed in density and expanded its range over time (Bischof et al., 2020; Sand et al., 2006). Based on a recent bear density map for the whole of Sweden (Bischof et al., 2020), we pooled the study sites into two groups: sites 1–5 as bear‐present areas (0–1.6 bears/km^2^, average 0.21 bears/km^2^) and site 6 as a bear‐free area (Figure 1).

Fennoscandian forest ecosystems are heavily managed with an extensive network of forest roads that increase accessibility (FAO, 2020; Flisberg et al., 2012; Forest Statistics, 2020; Fridholm, n.d.). Thus, wildlife has to adapt to human‐modified habitats, human presence, and infrastructure. Globally, Fennoscandia holds the highest densities of moose as well as the highest moose harvest rates (i.e., the annual harvest rate in Sweden is around 27% of the population, which reflected ~80,000 moose harvested per year during the last decade; Apollonio et al., 2010; Jensen et al., 2020). Harvest accounts for the major source of mortality in adult moose (Ericsson & Wallin, 2001), whereas large predators like brown bears affect calf survival during summer (Swenson et al., 2007). Wolves kill moose year‐round by targeting all categories (neonates‐adults; Sand et al., 2008). In our study, wolves occur at one site (nr 5, Figure 1) but only at low densities due to the recent expansion of the wolf population (Bischof et al., 2020; Sand et al., 2006). Like moose, large carnivores are subject to human harvest in Sweden as part of large carnivore management to balance different human interests (Swedish EPA, 2023). In Sweden, the annual bear hunt has existed since the 1980s after decades of hunting bans, which results in Scandinavian brown bears avoiding humans and their infrastructure (Frank et al., 2017; Moen et al., 2019; Nellemann et al., 2007; Ordiz et al., 2011).

### 2.2 Moose data

Female moose were immobilized with a CO_2_‐powered dart gun (DANiNJECT, Kolding, Denmark) from a helicopter with a mixture of etorphine‐acepromazine‐xylazine or etorphine‐xylazine (Evans et al., 2012; Græsli et al., 2020; Kreeger & Arnemo, 2007; Lian et al., 2014). Each female was equipped with a neck collar with a global positioning system (GPS) device, including a very high frequency (VHF) transmitter, a global system for mobile (GSM) communication, an ambient temperature recorder, and an acceleration sensor to monitor their movement over time (Vectronic Aerospace GmbH, Berlin, Germany, 2022). Using the GSM network or satellite, the tracking device sends continuous positions to the existing database Wireless Remote Animal Monitoring (WRAM; Dettki et al., 2014), which allows us to monitor females remotely in near real‐time. The GPS provided half‐hourly locations of the moose females, which we resampled for the habitat selection analysis to four times a day (00.00 h, 06.00 h, 12.00 h, and 18.00 h). Since moose are mostly active during dusk and dawn and females with calves move small distances during this period of the year (Neumann et al., 2012), we deemed four samples a day sufficient to capture female positions during both resting and active times to describe their major habitat selection. Moose are long‐lived, mostly solitary‐living, capital breeders with a life span of more than 20 years (Ericsson & Wallin, 2001). Females usually calve every year but their fertility decreases after the age of 15 years (Niedzialkowska et al., 2022). In our study, female age averaged 8.3 years (range 4–16 years) at their time of capture as indicated by their tooth wear (Ericsson & Wallin, 2001). All personnel handling the moose were certified according to the standards of the Swedish Animal Welfare Agency and the Swedish Board of Agriculture. The marking of moose has been approved by the Animal Care Committee in Umeå, Sweden (DNR A116‐09, A12‐12, A50‐12, A205‐12, A14‐15, A3‐16, A28‐17, A11‐2020).

GPS positions in near‐real time (Dettki et al., 2014) allow for monitoring of distinct changes in females' movement patterns during the calving season to remotely detect both parturition and early calf loss, which then can be verified in the field (Nicholson et al., 2019; Severud et al., 2015). During the calving season (May–June), we confirmed the number of calves and their status (i.e., alive or dead) for each moose female through field observations following patterns in the movement data, which indicated that the female had given birth (Neumann et al., 2020). We checked summer calf survival in the field before the annual moose hunt started in September/October (CAB, 2023). In between these two field controls, additional survival checks were performed when the movement of a given female indicated possible calf loss (i.e., sudden larger movement steps and return to the location of disturbance, Tallian et al., 2023). We confirmed predation of the calf by either finding calf remains with traces of predation using a dog and/or by seeing a distinct change in the female's movement pattern indicating predation (e.g., abrupt long steps covering several hundreds of meters, return to the calving site), and observing the female without a calf in the immediate next control (when remains were not found). Natural calf mortality does occur but only contributes up to 10% of their annual summer mortality (Swenson et al., 2007).

### 2.3 Data analysis

For each female, we linked movement data to her age and reproductive success in a given year (i.e., calf survival, calf loss due to predation or an unknown cause), resulting in a dataset including the calving date, the number of calves born, their summer survival, and the movement and age of the female. We marked adult moose at different ages, and we do not know about the experiences a given female had prior to the marking. We expect, however, that given the annual reproduction in moose, age would act as a proxy for accumulated reproductive experiences in female moose (Niedzialkowska et al., 2022). Survival of the calves during a given calving season was grouped into: “alive,” “predation,” or “other/unknown cause of death”. In this study, we were interested in the effect of experience with losing a calf on females' behavior: both calf loss in general and calf loss due to bear predation specifically. We considered calf loss as an experience event for the female and thus did not discriminate between losing one or two calves. When a female had two calves but lost one calf, we, therefore, assigned her to the group of females with at least one lost calf (i.e., either to the “predation group” or the group of other/unknown loss). When a female lost one calf due to predation and the other calf to another/unknown cause, we assigned the female to the predation group. We classified the disappearance of calves without any indication of predation as “other/unknown cause of death”. Calves can be stillborn or die immediately after birth due to complications or weakness (i.e., natural mortality). In our analyses, we excluded data from females with stillborn calves or calves that died immediately after birth as we assume these females had no time to establish any major relationship with their offspring, which may affect their anti‐predator behavior. We considered only females with data for the “calf survival in the previous year” and estimated age in our models, resulting in a total of 79 females and 162 female‐year combinations in the final dataset (Table 1).

**TABLE 1:** Number of females in areas with and without bears and their experiences with calf loss in the previous year in the final dataset.

Bear occurrence^a^	Calf survival in the previous year	Number of females	Number of year‐moose combinations
Bears (0–1.60 bears/km^2^, average 0.21 bears/km^2^)	Alive	34	61
	Predation	11	11
	Other/unknown	16	16
Bear‐free	Alive	28	60
	Predation	–	–
	Other/unknown	11	14
Total		100^a^	162

*Note*: The female moose had multiple years of data, resulting in multiple year‐moose combinations. Female moose (*n* = 79) can be in different calf survival groups in different years.

^a^ Estimates on bear densities in a given area as given by the raster of Bischof et al. (2020).

#### 2.3.1 Habitat selection during the calving season

To study the effect of the female experience on her selection for habitat structures at the calving site during the first 4 weeks following parturition in a given year, we built step selection functions using five random steps for each observed step and extracted habitat features at the end of the step to test for selection of a given habitat feature (R package “amt,” Signer et al., 2019). We based our decision to use five random steps on Thurfjell et al. (2014), who showed that as few as one random step per day is sufficient if one is not interested in the use of rare habitats. Moreover, a similar study by Van Beest et al. (2012) on moose habitat selection also used five random steps. Following the approach described in Signer et al. (2019), random steps were derived by fitting parametric distributions of both step length (gamma distribution) and turning angle (von Mises distribution). To analyze how the presence of bears affected the individual female selection of habitat structure and control for repeated measures and variation among females, we applied a conditional logistic regression using a generalized linear mixed model with a Poisson distribution (R package “glmmTMB,” Brooks et al., 2017; Muff et al., 2020). We used observed steps versus random steps as a dependent variable (binary). We used shrub cover, tree cover, terrain ruggedness and distance to the nearest road (all continuous), and bear presence and females' experience as independent variables (i.e., fixed effects) (Table 3). We assigned females' experience (i.e., fixed factor “calf survival in the previous year” (alive, predation, other/unknown cause of death)) and bear presence (bear‐free, bear‐present) as interaction terms with each habitat feature. Following the conditional logistic regression in a mixed model approach (Muff et al., 2020), we assigned female ID as random slope and step ID (one observed step and its linked five random steps) as random intercept, thereby accounting for autocorrelation, individual heterogeneity, repeated measures, and group‐specific stratum (Table 3).

We linked each moose position to a set of habitat features that describe habitat structure and vegetation occurrence at the given location. The structures we considered included: cover of low vegetation (as a proxy of shrub cover, vegetation 0.5–5 m as % of pixel covered), cover of high vegetation (as a proxy of tree coverage, vegetation 5–45 m as % of pixel covered), height of low vegetation (m, height interval per pixel), height of high vegetation (m, height interval per pixel), terrain ruggedness index and the Euclidean distance of the location to the nearest road (m; Table 2). We focused on these variables because they relate to calf concealment, predator detection or predator avoidance as well as a possible shield for predation risk (Bowyer et al., 1999; Nellemann et al., 2007; Severud et al., 2019). We checked for correlations between variables using Spearman rank correlation with a cut‐off value of *ρ* > .7. Vegetation height correlated strongly with vegetation cover for both vegetation types (low vegetation: *ρ* = .76, high vegetation: *ρ* = .82), and therefore we excluded vegetation height in our further analyses. The terrain ruggedness index infers differences in the elevation of a given pixel compared to the adjacent pixels on the map and is therefore a measure of topographic heterogeneity (Riley et al., 1999). The distance to the nearest roads as an index of human accessibility or presence was calculated as Euclidean distances in meters based on the Swedish road map including all roads (Trafikverket, 2014). We standardized all variables separately using the sample mean and sample standard deviation before adding them into the models to allow the comparison across sites.

**TABLE 2:** The habitat features with their year of collection, projection, resolution and source.

Variable	Year	Projection	Resolution	Source
Cover of low vegetation (%)	2018 Accessed 22‐02‐2022	SWEREF99 TM (EPSG:3006)	10 × 10 m	Swedish Environmental Protection Agency^a^
Cover of high vegetation (%)	2018 Accessed 22‐02‐2022	SWEREF99 TM (EPSG:3006)	10 × 10 m	Swedish Environmental Protection Agency^a^
Height of low vegetation (m)	2018 Accessed 22‐02‐2022	SWEREF99 TM (EPSG:3006)	10 × 10 m	Swedish Environmental Protection Agency^a^
Height of high vegetation (m)	2018 Accessed 22‐02‐2022	SWEREF99 TM (EPSG:3006)	10 × 10 m	Swedish Environmental Protection Agency^a^
Terrain ruggedness index	2009 Accessed 22‐02‐2022	RT90 2.5w (EPSG:2400)	50 × 50 m	Swedish Digital Elevation Model^b^
Distance to roads (m)	2014 Accessed 22‐09‐2015	SWEREF99 TM (EPSG:3006)	50 × 50 m	Swedish Transport Administration^c^

^a^ Naturvårdsverket (2020).

^b^ Lantmateriet (2009).

^c^ Trafikverket (2014).

Calf vulnerability to bear predation decreases sharply with time (Swenson et al., 2007), as calf mobility increases (Testa et al., 2000). In our data, predation events decreased clearly after the second week of the calves' lifetime. To account for this changing risk of predation, we divided our data into four groups: birth (calving date), first week (1–7 days after birth), second week (8–14 days after birth), and later in the calving season (third/fourth week; 15–28 days after birth). To avoid adding too many interaction terms, we made separate models for these different groups. In habitat selection modeling, the relative risk (exp(coef)) is interpreted as the relative strength of selection (i.e., RSS; Avgar et al., 2017), which we used to determine the selection for habitat features in relation to each other using a full model approach (i.e., including all fixed and random effects as listed above). We extracted the estimates for the main effects of the habitat variables (averaged over bear presence and female experience) from the model summary (Appendix S1). Next, we determined the estimates for each combination of habitat variable with bear presence or female experience using estimated marginal means of linear trends (emtrends, R package “emmeans,” Lenth, 2023). For all estimates, we calculated the exponential values, which inform on which habitat features female moose select or avoid (i.e., RSS values; original estimates inform on selection strength only). To highlight differences in females' selection behavior over time, we calculated RSS values derived from each model separately but presented them together in the figures.

#### 2.3.2 Site fidelity during the calving season

To test for site fidelity of female moose in relation to bear presence and females' experience among years, we modeled the observed inter‐annual distances between calving site locations in successive years calculated as Euclidean distances (km) separately for each female. For each female, we calculated this distance as the distance between the daily average GPS collar locations in successive years, for each day, starting from the birth date (day 0) through the end of the first week after calving (day 7). This allowed us to compare female selection (i.e., derived by the coordinates) both at the date of calving and during the first week among successive years.

We tested for differences in distances among calving sites between years in relation to bear presence and female experience using a linear mixed model. We applied the distance between sites (cube root transformed to ensure normality) as the dependent variable and the fixed effects “calf survival in the previous year” (alive, predation, other/unknown cause of death), bear presence (bear‐free, bear‐present) and female age as independent variables (R package “nlme”, Pinheiro & Bates, 2000, Table 3). We included the female ID as a random intercept to account for autocorrelation, individual heterogeneity, and repeated measures (Pinheiro & Bates, 2000). To account for the effect of age and the possibility of accumulated experiences in relation to females' site fidelity of calving sites, we added age as a fixed effect. We applied two models: one on the birth sites (i.e., calving dates) and one on the first week after calving, thereby estimating site fidelity for the calving site itself as well as for the area utilized during the most vulnerable period for the calf. We applied ANOVA (type = marginal) and Tukey pairwise comparisons to analyze the effects of different factors in the models.

**TABLE 3:** (Generalized) linear mixed models to test the feature selection of moose during the first 4 weeks after calving using a step selection function (1) and to test for site fidelity (2).

Research question	Fixed effects	Model	Data
(1) Feature selections	Case^a^ ~ −1 + shrub cover^b^ + tree cover^c^ + terrain ruggedness^d^ + road^e^ + (shrub cover^b^ + tree cover^c^ + terrain ruggedness^d^ + road^e^): (calf survival in previous year^f^ + bears^g^) + (1|step ID) + (0 + shrub cover^b^|female ID) + (0 + tree cover^c^|female ID) + (0 + terrain ruggedness^d^|female ID) + (0 + road^e^|female ID)	Conditional generalized linear mixed model (glmmTMB) with a Poisson distribution	Separately for each after birth (calving date, week 1, week 2, weeks 3 + 4)
(2) Site fidelity	Distance ~ age^h^ + bears^g^ + calf survival in the previous year^f^ + (1|female ID)	Linear mixed model	Separately for the calving date and the first week after birth

*Note*: The site selection model includes main effects and interaction effects. In the conditional logistic regression, we assigned female ID to allow for individual‐specific random slopes, but omitted the random intercepts following Muff et al. (2020). In the linear regression, we included female ID as individual‐specific random intercepts (Pinheiro & Bates, 2000).

^a^ Observed and random steps; binary.

^b^ Cover of low (0.5–5 m) vegetation (%); continuous.

^c^ Cover of high (5–45 m) vegetation (%); continuous.

^d^ Terrain ruggedness index; continuous.

^e^ Euclidean distance to the nearest road (m); continuous.

^f^ Alive, lost due to bear predation or lost due to other/unknown cause; categorical.

^g^ Bear‐free, bears present; categorical.

^h^ Female age; continuous.

All spatial and statistical analyses were performed in R – version 4.3.1 (R Core Team, 2023), with a significance level of *p* < .05.

## 3 RESULTS

We analyzed movement data of 79 adult free‐ranging female moose (average age 8 years, ranging from 4 to 16 years) and associated data on the survival of their calves over multiple years (2013–2021) during the calving season in six study sites located across Sweden. We analyzed a total of 18,143 observed movement steps (each step builds a group stratum of one observed and five random steps, resulting in a final total of 108,858 steps). The average number of observed steps per female was 230 steps (ranging from 75 to 569 steps). For 73 females, we could analyze the site fidelity of calving sites in consecutive years.

### 3.1 Female selection for habitat structure in relation to bear presence and individual experiences with bears

**FIGURE 2:**
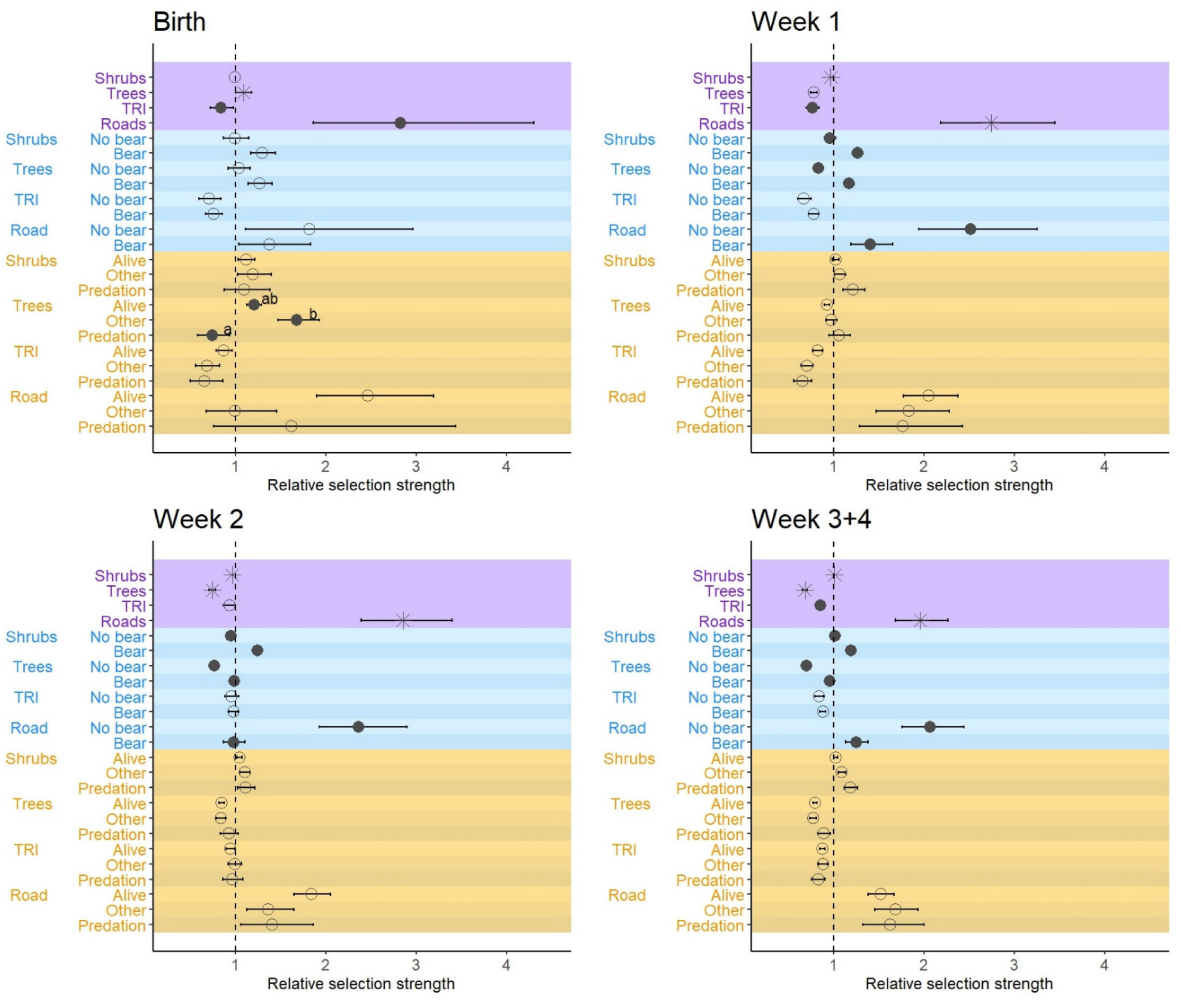
The relative selection strength for different habitat features by female moose (*n* = 79) in relation to bear presence (yes/no) and individual experience the previous year (i.e., calf survival) during the first 4 weeks following parturition, Sweden 2013–2021. We analyzed female response at the calving date, during the first week after parturition, the second week after parturition and for the combination of the third and fourth week after parturition. The plots show the estimated main effects of the model, which are averaged over the bear presence and individuals experience categories (purple), the effect of bear presence (blue) and the effect of individual experiences (orange). Significance (*p* < .05; filled dots) indicates evidence for differences between the relevant groups (i.e., bear vs. no bear, alive vs. other vs. predation). *At the main effects indicate that both the main effect and at least one interaction is significant. Selection for lower values compared to the random locations is on the left side of the vertical dashed line and selection for higher values is on the right side. The horizontal lines show the standard errors. Shrubs = shrub cover, Trees = tree cover, TRI = terrain ruggedness, Road = Euclidean distance to roads. The annotations (a, ab, b) show which variables are significantly different from one another (different letter) or not significantly different (same letter).

Bear presence affected females' selection for habitat features at the calving site during the weeks following parturition, whereas individual experience affected their selection at the calving date (Figure 2; Appendix S1 and S2). In bear areas, females selected more strongly for high percentages of shrub cover compared to females in bear‐free areas during the weeks following parturition (i.e., weeks 1–4). Females in the bear areas reduced their selection for tree cover over time (i.e., from high to lower percentages of cover, weeks 1–4). Females in the bear areas selected less strongly for greater distances to the nearest road compared to the females in the bear‐free area (weeks 1–4).

At the calving date, the selection for cover differed with females' experiences. Females with bear‐predated calves in the previous year selected for locations with lower tree cover compared to females with calves lost to other/unknown reasons (Figure 2). We did not find any evidence that females with surviving or bear‐killed calves differed in selection for shrub cover, terrain ruggedness, or distance to roads for any of the weeks or the calving date. Looking at the effect of one given habitat feature at a time, we found some variation among predictors and time in relation to the bear presence and females' individual experience, particularly at the calving date and during the first week after calving (e.g., the effect of tree cover). In general, variation decreased during the following weeks (i.e., weeks 2–4), such as for ruggedness, tree cover, and distance to roads in bear areas (Appendix S3).

### 3.2 Female calving site fidelity in relation to bear occurrence and experience

**FIGURE 3:**
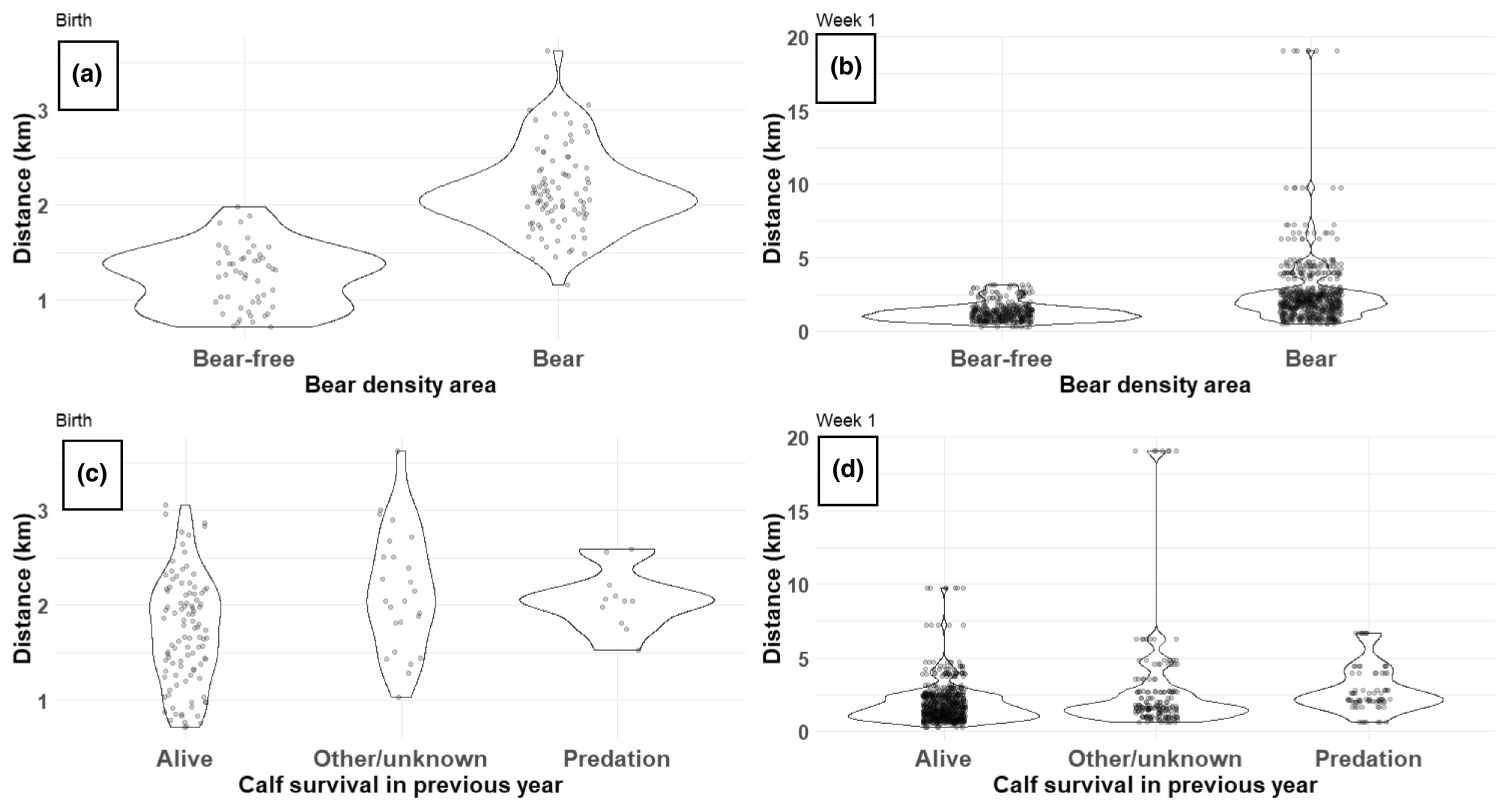
The effect of bear presence (a, b) and individual experiences (i.e., calf survival) (c, d) on the distance between successive calving sites of female moose (*n* = 73) in Sweden, 2013–2021. We made separate models for the calving date (a, c) and the first week after calving (b, d), with bear presence and calf survival included in the same model. The areas were either bear‐free or had bears. The female moose had either calves that survived the previous year, were lost due to other/unknown reasons or were lost due to bear predation.

At the calving date (i.e., the calving site itself), bear presence influenced the site fidelity in female moose (*F*_1,71_ = 3.8, *p* = .05). In the bear‐free area, females calved on average closer to their calving sites of the previous year (mean distance = 1.4 km) compared to females in areas with bears (mean distance = 2.1; Figure 3a). On the other hand, we did not find any evidence that calf survival or loss in the previous year (i.e., females' experience) or female age influenced the distance between subsequent calving sites (survival: *F*_2,62_ = 0.4, *p* = .64; age: *F*_1,62_ = 1.3, *p* = .26; Figure 3c).

During the first week after calving (i.e., the area the female utilized shortly after parturition when the calf is most vulnerable to predation), both bear presence and individual experience (i.e., calf survival in the previous year) influenced females' site fidelity (bear presence: *F*_1,71_ = 12.8, *p* < .001; survival: *F*_2,891_ = 6.2, *p* = .002). Similar to the calving date, female moose in the bear‐free area showed a stronger site fidelity (i.e., higher proximity) between the place they used during the first week after parturition the previous year compared to females in bear areas (Figure 3b; bear‐free: mean = 1.2 km; bear: mean = 2.6 km, *t*_71_ = −3.6, *p* < .001). In addition, females with surviving calves in the previous year had a higher site fidelity (i.e., smaller distance between sites) compared to females that lost their calves in the previous year due to other/unknown causes. However, we did not find evidence for females with predated calves having a different site fidelity from that of females with surviving calves (surviving: mean = 1.5 km; other/unknown: mean = 2.0 km, *t*_891_ = − 3.3, *p* = .003; predation: mean = 2.0 km, *t*_891_ = −1.8, *p* = .18; Figure 3d). We did find evidence for an effect of females' age (*F*_1,891_ = 10.2, *p* = .002), with older females showing on average a weaker site fidelity than younger ones (*t*_891_ = 3.2, *p* = .002).

## 4 DISCUSSION

Our study on calving site selection and site fidelity in a long‐lived ungulate species suggests two points. First, our findings indicate that females responded to the presence of bears during the calving period, i.e., females in different areas with bears selected for other habitat features than females in the bear‐free area. More specifically, our results suggest that females in areas with bears selected for habitat features connected to lower bear encounter risk and showed lower site fidelity compared to females in the bear‐free area. Second, although bear presence influences females' selection in the weeks following parturition, our results suggest that the individual experience of a calf loss (e.g., the risk of a calf being killed during a bear encounter) alters a female's selection during the calving day the following year. The observed behavioral differences we found between females that experienced calf loss and those that did not suggest that the individual experience of losing a calf may result in a learned response (i.e., changed selection of habitat features) to reduce calves' mortality risk (e.g., calves become killed when encountering a bear in bear areas). This behavioral change following individual experience indicates behavioral plasticity in a long‐lived and solitary living ungulate species.

In long‐lived species that reproduce regularly, such as many ungulate species, age can play an important role in how a given individual responds to different stimuli, such as mortality risk in general or the risk of predation specifically (Blank, 2018; Lima, 1992; Lima & Dill, 1990; Thurfjell et al., 2017). Yet, the effect of age may also be context‐ and species‐specific (e.g., site fidelity in relation to age in different ungulate species, Morrison et al., 2021). Our findings indicate that the individual experience of a calf loss affects the behavior the following year in a long‐lived and solitary living ungulate species like moose, leading older females (and thus likely more experienced individuals) to adjust their choice of calving site over time, which in turn may reduce the mortality risk for their offspring (Lima & Dill, 1990). We found an effect of female age on site fidelity in the first week after parturition but not at the calving date itself. This might be that for the birth date, data variation might be larger than for the entire week as – depending on the exact timing of the parturition – the birth date can also include positions just prior to the calf being born. We did not find any quality differences (as indicated by positions' dilution of precision (DOP)) between the two periods.

For large‐bodied herbivores, selection for more tree and shrub cover following parturition can provide both calf concealment and forage (Bongi et al., 2008; Ciuti et al., 2005; Severud et al., 2019). For example, fallow deer fawns select bedding sites with higher amounts of canopy cover compared to random sites (Kjellander et al., 2012), and roe deer shift from open to forested areas during the fawning season (Bongi et al., 2008). Cover might be particularly important for solitary browsing species as females cannot rely on shielding from other reproducing conspecifics but can utilize the forage produced by shrubs (le Roux et al., 2009; Tablado et al., 2016). Selection for shrub cover, however, might come with trade‐offs. On one hand, shrub cover may protect from predators visually (White & Berger, 2001) and may reduce smell for predators like bears that use olfactory cues, but may also allow a predator to approach undetected. Tree cover can also provide thermal shelter and forage, which is relevant for heat‐sensitive ungulates like moose with increasing ambient temperatures (van Beest et al., 2012). However, our findings were inconclusive for this argument as we found females in the bear‐free and southernmost study area (with higher average ambient temperatures; SMHI, 2017) selecting for less tree cover. Our results, therefore, did not support the idea that tree cover is selected for thermal shelter during critical periods of the calving season in our study settings. Given the fact that females in the bear areas selected for higher shrub and tree cover than females in the bear‐free area, we suggest that utilizing vegetation cover might help to camouflage calves in the presence of bears as a strategy to reduce the encounter risk with bears during the calving season, which is in line with previous research (Bowyer et al., 1999).

We found no evidence that bears presence prompts moose mothers to alter their selection of rugged habitat features during the calving season. However, averaged over all moose, we found a selection for less rugged terrain. Terrain ruggedness provides the possibility for prey to place themselves on elevated spots compared to the surroundings, thereby increasing the chance of picking up olfactory, visual, and auditory cues from approaching predators such as bears (Bowyer et al., 1999; Wilton & Garner, 1991). As a result, rugged terrain may help to reduce the encounter risk as well as mortality risk for neonates given a predator encounter. This might be especially important for prey with good olfactory and auditory senses like ungulates. In ecosystems where predators select for terrain ruggedness, prey might choose to select against the positive attributes that come with terrain ruggedness. In Scandinavia, bears prefer rugged forested terrain because it usually comes with lower disturbance by humans as well as rugged terrain supports the availability of (food) plants and denning sites (Nellemann et al., 2007). Thus, within the Scandinavian context, for moose, this might result in sensitive trade‐offs between positioning themselves at spots that provide more information but also may come with a larger risk of encountering a bear. We suggest more in‐depth research on terrain ruggedness selection as anti‐predator behavior of ungulates in anthropogenic landscapes with bears where both predator and prey are subject to human harvest.

In human‐altered landscapes, infrastructure objects can generate predator‐free refuges for prey if predators avoid humans (Muhly et al., 2011). For example, distance to the nearest road can act as a refuge feature in Yellowstone National Park, USA, as moose select birth sites closer to roads, away from road‐avoiding bears (Berger, 2007). Our work did not support this finding in our anthropogenic system. By contrast, − except for the calving day itself, during which the moose neither selected nor avoided habitats based on distance to roads – we found females consistently selected habitats farther from roads. However, the strength of this selection was much stronger in females in the bear‐free area compared to females in bear areas. In May and June, increased bear sightings along/across roads occur in Scandinavia (personal comments: Stenbacka and Neumann), suggesting that bears occur close to roads during the moose calving season. This period overlaps largely with the bear mating season (García‐Rodríguez et al., 2020), where bears show higher activity and larger space use within their home ranges (Dahle & Swenson, 2003), which may come along with the crossing of more roads. In Northern Scandinavia, where bears occur, June–July is also the period of 24‐h daylight (i.e., midsummer), which largely increases visibility and thus likely detectability and observation rates of bears. Because the utilization of areas around roads by both predator and prey is influenced by multiple factors (Neumann et al., 2013; Ordiz et al., 2014), we recommend further research on the effect of roads on predator–prey interactions.

Ungulates use calving site fidelity as an anti‐predator strategy and this strategy varies with females’ experience. For example, site fidelity of both Alaskan moose and caribou (*Rangifer tarandus caribou*) was higher for females with surviving calves in the previous year, and in the case of moose, it was enough if at least one calf survived (Testa et al., 2000; Wittmer et al., 2006). We found that site fidelity was lower for females in bear areas compared to the bear‐free area, regardless of whether the female had experienced calf loss or not. This finding indicates that females in bear areas expressed behavior that makes their calving site placement less predictable, which in turn may lower the encounter risk with a bear. Site fidelity is complex and context‐specific (e.g., species, season, environment; Morrison et al., 2021). Concerning fidelity to a given calving site, our results are in line with previous research suggesting that positive experiences affect site fidelity (i.e., surviving calves, Testa et al., 2000; Wittmer et al., 2006). However, it is important to note that although we found a clear effect of calf loss of other/unknown causes and survival, we found no effect of experienced calf predation in the previous year. This lack of evidence might be due to the small sample of confirmed bear predations in our study. Therefore, we suggest future research should increase the number of sampling years in bear‐dense areas to be better able to discriminate among the three levels of experience (i.e., surviving calves, lost due to other/unknown causes and lost due to predation).

We used a comprehensive dataset on position and calf survival data in different areas to test for the effect of bear presence on females' selection for calving sites and site fidelity. A few limitations need mentioning. First, in our analyses, we compared several bear areas to one bear‐free area. Thus, an area‐specific response might have influenced our results. We therefore recommend future research to include more replicates if available. Second, as this study completely focused on bears as the major predator of newborn calves during their first weeks of life (Swenson et al., 2007), we did not consider the possible effect of wolves despite wolves having re‐colonized the areas during recent years (Bischof et al., 2020). To improve our understanding of the effect of multiple carnivore species on ungulates during the calving season and possible trade‐offs in anti‐predator behavior (Leblond et al., 2016), we encourage future research to include wolves to test if the effect of predator presence and females' individual experiences may differ for these two predators in female ungulates. In the anthropogenic landscapes of Europe, wolves are returning (Chapron et al., 2014), including areas where brown bears have dominated during the past decades (Ordiz et al., 2015, e.g., site nr 5 in our study). This may challenge prey to adjust their anti‐predator strategies accordingly. We are, however, aware of the practical challenge such a study may involve as it asks for a sufficient sample size of both verified bear‐ and wolf‐killed calves. Next, in human‐dominated systems, similar research may also be applied to human hunting pressure on ungulates to see if long‐lived and solitary prey species like moose can express predator‐specific responses during times of high mortality risk given by bears, wolves or humans. We know that ungulates adjust their behavior to different types of mortality risk in relation to experiences (e.g., harvest and wolves; Graf, 2021; Proffitt et al., 2009; Thurfjell et al., 2017), but we still lack a comprehensive understanding of which experiences trigger which behavioral responses to given mortality risk in the same system. Personality affects individuals' responses to perceived risk and thus their choice of certain habitat features (Brehm & Mortelliti, 2021). Together with learning events, personality can generate specific behavioral patterns in habitat selection in relation to mortality risk, thereby explaining variation across individuals (Ciuti et al., 2012; Graf, 2021; Thurfjell et al., 2017). Including personality aspects in the analyses was beyond the scope of this study. Yet, given the variation among individual females and to get a comprehensive understanding of the behavioral responses to perceived and experienced risk, we suggest future research to address whether certain individuals respond consistently more conservatively than others do. Moreover, future research with access to a sufficient sample size should study whether losing twins results in different and/or stronger responses than losing one calf. Last, in this study, we focused on possible strategies a female ungulate species applies during the calving season derived from their previous experience, but it did not look at the success of a given strategy. A natural follow‐up study is to link females' site selection and site fidelity to the likelihood of increasing calf survival in relation to females' experiences in the previous year.

## 5 CONCLUSIONS AND IMPLICATIONS

Our study highlights how predator presence (i.e., bear presence) and individual experiences (i.e., experience with previous calf survival or loss) shape both the selection of habitat features at the calving site and site fidelity in a long‐lived solitary ungulate like moose, suggesting that the expressed behavior is a response to the risk of encountering bears and the calf mortality risk. We found evidence for responses to bear presence and experienced calf loss. Our work particularly not only emphasizes the importance of shrub cover but also of tree cover, for the female moose in relation to bear presence during the calving season. Therefore, we recommend both forestry and wildlife habitat management to ensure the availability of suitable cover of low (i.e., shrubs) as well as high vegetation during the calving season for browsing herbivores like moose in areas with bears. Moreover, in areas with extensive road networks and relatively high human activity as in many places in Europe, roads might generate interesting effects in prey–predator interactions that might differ from less human‐dominated systems. In systems where both prey and predators are subject to human harvest, infrastructure like roads might affect both prey and predators' space use patterns of near‐road habitats differently for different reasons and times (Bischof et al., 2016; Neumann et al., 2013; Ordiz et al., 2014), which should be studied and considered further in prey–predator studies. With returning predators or increasing predator densities, these habitat structures, including anthropogenic features, may become increasingly important to ensure reproductive success and thus sustainable recruitment numbers. With this study, we highlight different strategies of female moose in relation to bear presence and individual experience, but the effect of the observed selection strategies on reproductive success was beyond the scope of this study. We, therefore, recommend future research to follow up on our study, including the consideration of the effectiveness of the applied strategies.

## AUTHOR CONTRIBUTIONS

**Lisa Dijkgraaf:** Formal analysis (lead); investigation (lead); methodology (equal); software (lead); validation (equal); visualization (lead); writing – original draft (lead); writing – review and editing (lead). **Fredrik Stenbacka:** Data curation (equal); formal analysis (supporting); methodology (supporting); resources (equal); supervision (supporting); validation (equal); writing – original draft (equal); writing – review and editing (equal). **Joris P. G. M. Cromsigt:** Methodology (supporting); validation (equal); writing – original draft (equal); writing – review and editing (equal). **Göran Ericsson:** Funding acquisition (lead); project administration (lead); resources (lead); validation (equal); writing – original draft (equal); writing – review and editing (equal). **Wiebke Neumann:** Conceptualization (lead); data curation (equal); formal analysis (supporting); investigation (equal); methodology (lead); project administration (equal); resources (equal); software (equal); supervision (lead); validation (lead); visualization (supporting); writing – original draft (equal); writing – review and editing (equal).

## Supporting information


Appendix S1

## Data Availability

Data and R code are available at Neumann et al. (2024). Dryad. https://doi.org/10.5061/dryad.m0cfxpp9t.
