# Supplementary material for: Bear in mind! Bear presence and individual experience with calf survival shape the selection of calving sites in a long‐lived solitary ungulate
Source: Ecol Evol. 2024 Mar 19;14(3):e11177. doi: 10.1002/ece3.11177 (PMC10950790; doi:10.1002/ece3.11177)
Supplement: Supplementary file 1 — Appendix S1 [file ECE3-14-e11177-s001.docx]

**Appendices**

Bear in mind! Bear presence and individual experience with calf survival shape the selection of calving sites in a long-lived solitary ungulate

Lisa Dijkgraaf ^1,2^, Fredrik Stenbacka^2^, Joris PGM Cromsigt^2^, Göran Ericsson^2^, Wiebke Neumann^2^

^1^ Department of Wildlife Ecology and Conservation, Wageningen University (WUR)

^2^ Department of Wildlife, Fish, and Environmental Studies, Swedish University of Agricultural Sciences (SLU)

**Appendix S1**. The marginal relative selection strength for different habitat features by female moose (n=79) in relation to bear presence and individual experiences during the first four weeks following parturition as given by the conditional logistic mixed regression, Sweden 2013-2021. The calculation of the coefficient sum indicates the marginal selection strength by a given combination of the fixed effects (i.e. bear presence and calf survival) for a given habitat feature. No difference between observed and random step is indicated by the dashed vertical line. Values on left of side of the dashed line indicate females’ selection for lower values compared to availability (i.e. the random steps), values on the right side selection for higher values . Shrub = shrub cover, Tree = tree cover, TRI = terrain ruggedness, Road = Euclidean distance to roads.

**
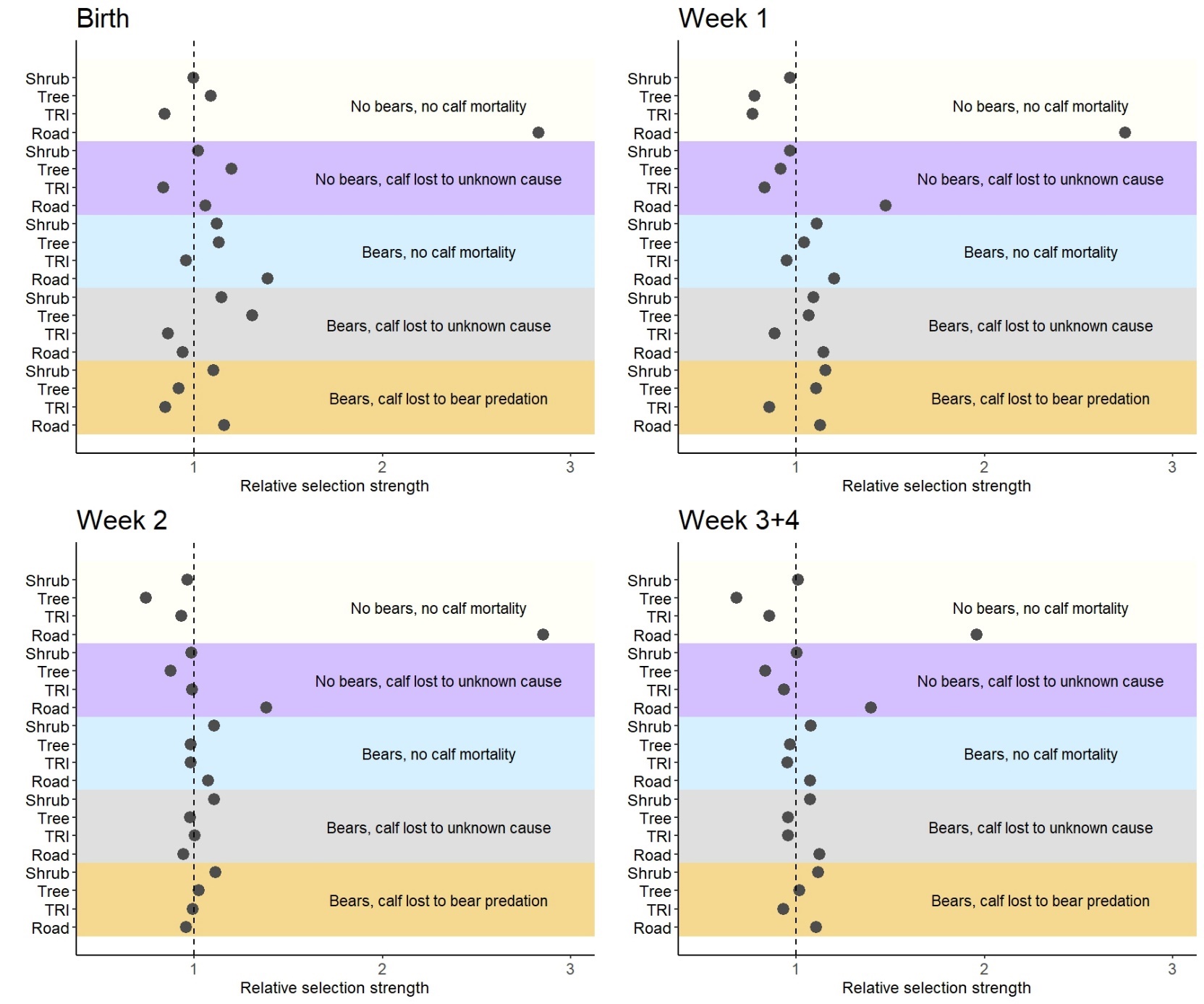
**

**Appendix S2**. The estimates of marginal relative selection strength for different habitat features by female moose (n=79) in relation to bear presence and individual experiences during the first four weeks following parturition as given by the conditional logistic mixed regression for a given time, Sweden 2013-2021. Shrub = shrub cover, Tree = tree cover, TRI = terrain ruggedness, Road = Euclidean distance to roads.

| Calving date |  |  |  |  |
| --- | --- | --- | --- | --- |
| Habitat | Category | Estimate | SE | RSS |
| Shrub |  | -0.001 | NaN | 0.999 |
| Tree |  | 0.086 | 0.080 | 1.090 |
| TRI |  | -0.168 | 0.152 | 0.845 |
| Road |  | 1.040 | 0.419 | 2.829 |
| Shrub | Bear-free | -0.002 | 0.140 | 0.998 |
| Shrub | Bear | 0.262 | 0.104 | 1.300 |
| Tree | Bear-free | 0.036 | 0.116 | 1.037 |
| Tree | Bear | 0.238 | 0.102 | 1.269 |
| TRI | Bear-free | -0.343 | 0.174 | 0.710 |
| TRI | Bear | -0.276 | 0.120 | 0.759 |
| Road | Bear-free | 0.599 | 0.489 | 1.820 |
| Road | Bear | 0.321 | 0.284 | 1.379 |
| Shrub | Alive | 0.114 | 0.082 | 1.121 |
| Shrub | Other/unknown | 0.180 | 0.157 | 1.197 |
| Shrub | Predation | 0.097 | 0.230 | 1.102 |
| Tree | Alive | 0.187 | 0.068 | 1.206 |
| Tree | Other/unknown | 0.521 | 0.135 | 1.683 |
| Tree | Predation | -0.296 | 0.235 | 0.744 |
| TRI | Alive | -0.134 | 0.099 | 0.874 |
| TRI | Other/unknown | -0.378 | 0.191 | 0.685 |
| TRI | Predation | -0.416 | 0.271 | 0.660 |
| Road | Alive | 0.901 | 0.261 | 2.462 |
| Road | Other/unknown | -0.004 | 0.384 | 0.996 |
| Road | Predation | 0.483 | 0.751 | 1.621 |

| Week 1 |  |  |  |  |
| --- | --- | --- | --- | --- |
| Habitat | Category | Estimate | SE | RSS |
| Shrub |  | -0.030 | 491.336 | 0.970486 |
| Tree |  | -0.246 | 0.047 | 0.781986 |
| TRI |  | -0.262 | 0.088 | 0.76942 |
| Road |  | 1.010 | 0.228 | 2.746465 |
| Shrub | Bear-free | -0.040 | 0.063 | 0.960842 |
| Shrub | Bear | 0.235 | 0.046 | 1.264478 |
| Tree | Bear-free | -0.185 | 0.061 | 0.830935 |
| Tree | Bear | 0.161 | 0.049 | 1.174185 |
| TRI | Bear-free | -0.391 | 0.106 | 0.67653 |
| TRI | Bear | -0.248 | 0.073 | 0.780546 |
| Road | Bear-free | 0.922 | 0.258 | 2.514915 |
| Road | Bear | 0.340 | 0.164 | 1.405379 |
| Shrub | Alive | 0.026 | 0.036 | 1.026511 |
| Shrub | Other/unknown | 0.067 | 0.060 | 1.069455 |
| Shrub | Predation | 0.199 | 0.099 | 1.219885 |
| Tree | Alive | -0.073 | 0.034 | 0.929574 |
| Tree | Other/unknown | -0.022 | 0.058 | 0.977802 |
| Tree | Predation | 0.059 | 0.111 | 1.06028 |
| TRI | Alive | -0.191 | 0.059 | 0.826455 |
| TRI | Other/unknown | -0.345 | 0.093 | 0.70852 |
| TRI | Predation | -0.423 | 0.151 | 0.655326 |
| Road | Alive | 0.719 | 0.146 | 2.053095 |
| Road | Other/unknown | 0.605 | 0.219 | 1.830843 |
| Road | Predation | 0.570 | 0.317 | 1.767725 |
|  |  |  |  |  |
|  |  |  |  |  |
|  |  |  |  |  |
|  |  |  |  |  |
| Week 2 |  |  |  |  |
| Habitat | Category | Estimate | SE | RSS |
| Shrub |  | -0.036 | NaN | 0.964 |
| Tree |  | -0.294 | 0.049 | 0.745 |
| TRI |  | -0.068 | 0.071 | 0.934 |
| Road |  | 1.048 | 0.176 | 2.853 |
| Shrub | Bear-free | -0.049 | 0.050 | 0.953 |
| Shrub | Bear | 0.222 | 0.037 | 1.249 |
| Tree | Bear-free | -0.267 | 0.062 | 0.766 |
| Tree | Bear | -0.008 | 0.049 | 0.992 |
| TRI | Bear-free | -0.044 | 0.081 | 0.957 |
| TRI | Bear | -0.018 | 0.052 | 0.982 |
| Road | Bear-free | 0.859 | 0.204 | 2.362 |
| Road | Bear | -0.020 | 0.122 | 0.980 |
| Shrub | Alive | 0.049 | 0.027 | 1.050 |
| Shrub | Other/unknown | 0.101 | 0.052 | 1.107 |
| Shrub | Predation | 0.111 | 0.086 | 1.117 |
| Tree | Alive | -0.164 | 0.035 | 0.848 |
| Tree | Other/unknown | -0.174 | 0.061 | 0.840 |
| Tree | Predation | -0.074 | 0.107 | 0.929 |
| TRI | Alive | -0.055 | 0.049 | 0.946 |
| TRI | Other/unknown | -0.007 | 0.074 | 0.993 |
| TRI | Predation | -0.030 | 0.115 | 0.970 |
| Road | Alive | 0.609 | 0.109 | 1.838 |
| Road | Other/unknown | 0.311 | 0.191 | 1.365 |
| Road | Predation | 0.340 | 0.281 | 1.405 |

| Week 3+4 |  |  |  |  |
| --- | --- | --- | --- | --- |
| Habitat | Category | Estimate | SE | RSS |
| Shrub |  | 0.013 | 28023.540 | 1.013 |
| Tree |  | -0.380 | 0.040 | 0.684 |
| TRI |  | -0.152 | 0.051 | 0.859 |
| Road |  | 0.672 | 0.148 | 1.959 |
| Shrub | Bear-free | 0.017 | 0.039 | 1.017 |
| Shrub | Bear | 0.177 | 0.028 | 1.194 |
| Tree | Bear-free | -0.352 | 0.049 | 0.703 |
| Tree | Bear | -0.045 | 0.037 | 0.956 |
| TRI | Bear-free | -0.169 | 0.059 | 0.845 |
| TRI | Bear | -0.124 | 0.039 | 0.883 |
| Road | Bear-free | 0.728 | 0.164 | 2.071 |
| Road | Bear | 0.225 | 0.100 | 1.252 |
| Shrub | Alive | 0.027 | 0.022 | 1.027 |
| Shrub | Other/unknown | 0.091 | 0.038 | 1.095 |
| Shrub | Predation | 0.174 | 0.061 | 1.190 |
| Tree | Alive | -0.226 | 0.028 | 0.797 |
| Tree | Other/unknown | -0.256 | 0.046 | 0.774 |
| Tree | Predation | -0.113 | 0.080 | 0.893 |
| TRI | Alive | -0.130 | 0.034 | 0.878 |
| TRI | Other/unknown | -0.124 | 0.058 | 0.883 |
| TRI | Predation | -0.185 | 0.085 | 0.831 |
| Road | Alive | 0.421 | 0.094 | 1.523 |
| Road | Other/unknown | 0.521 | 0.141 | 1.683 |
| Road | Predation | 0.487 | 0.206 | 1.628 |

**Appendix S3.** Prediction plots on the calculated effect of a given habitat feature on female moose habitat selection while holding all other predictors in the model constant (mean value). The models are done separately for each week of the first four weeks following parturition. Sweden 2013-2021.

**
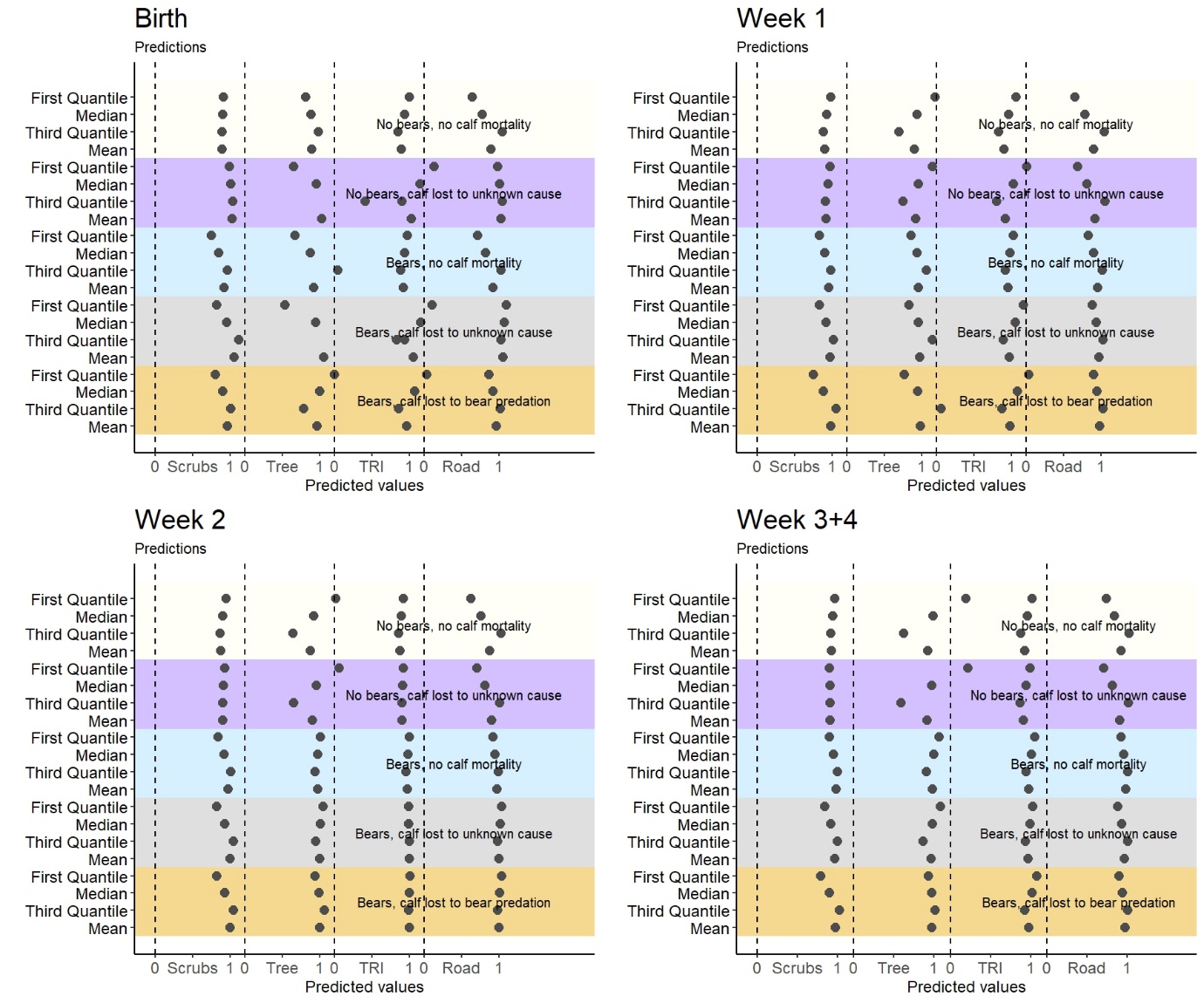
**
